# Amyloid Beta Immunoreactivity in the Retinal Ganglion Cell Layer of the Alzheimer’s Eye

**DOI:** 10.3389/fnins.2020.00758

**Published:** 2020-07-31

**Authors:** Sieun Lee, Kailun Jiang, Brandon McIlmoyle, Eleanor To, Qinyuan (Alis) Xu, Veronica Hirsch-Reinshagen, Ian R. Mackenzie, Ging-Yuek R. Hsiung, Brennan D. Eadie, Marinko V. Sarunic, Mirza Faisal Beg, Jing Z. Cui, Joanne A. Matsubara

**Affiliations:** ^1^Department of Ophthalmology and Visual Sciences, Faculty of Medicine, The University of British Columbia, Vancouver, BC, Canada; ^2^School of Engineering Science, Simon Fraser University, Burnaby, BC, Canada; ^3^Department of Surgery, Division of Ophthalmology, University of Calgary, Calgary, AB, Canada; ^4^Department of Family Medicine, Queen’s University, Kingston, ON, Canada; ^5^Department of Pathology, Vancouver General Hospital, The University of British Columbia, Vancouver, BC, Canada; ^6^Division of Neurology, Department of Medicine, Faculty of Medicine, The University of British Columbia, Vancouver, BC, Canada; ^7^Department of Ophthalmology and Visual Sciences, Dalhousie University, Halifax, NS, Canada

**Keywords:** Alzheimer’s disease, retina, amyloid-beta, Temporal retina, Neuritic plaques, ophthalmic imaging, mid-peripheral retina, retinal ganglion cell

## Abstract

Alzheimer’s disease (AD) is the most prevalent form of dementia, accounting for 60–70% of all dementias. AD is often under-diagnosed and recognized only at a later, more advanced stage, and this delay in diagnosis has been suggested as a contributing factor in the numerous unsuccessful AD treatment trials. Although there is no known cure for AD, early diagnosis is important for disease management and care. A hallmark of AD is the deposition of amyloid-β (Aβ)-containing senile neuritic plaques and neurofibrillary tangles composed of hyperphosporylated tau in the brain. However, current *in vivo* methods to quantify Aβ in the brain are invasive, requiring radioactive tracers and positron emission tomography. Toward development of alternative methods to assess AD progression, we focus on the retinal manifestation of AD pathology. The retina is an extension of the central nervous system uniquely accessible to light-based, non-invasive ophthalmic imaging. However, earlier studies in human retina indicate that the literature is divided on the presence of Aβ in the AD retina. To help resolve this disparity, this study assessed retinal tissues from neuropathologically confirmed AD cases to determine the regional distribution of Aβ in retinal wholemounts and to inform on future retinal image studies targeting Aβ. Concurrent post-mortem brain tissues were also collected. Neuropathological cortical assessments including neuritic plaque (NP) scores and cerebral amyloid angiopathy (CAA) were correlated with retinal Aβ using immunohistochemistry, confocal microscopy, and quantitative image analysis. Aβ load was compared between AD and control (non-AD) eyes. Our results indicate that levels of intracellular and extracellular Aβ retinal deposits were significantly higher in AD than controls. Mid-peripheral Aβ levels were greater than central retina in both AD and control eyes. In AD retina, higher intracellular Aβ was associated with lower NP score, while higher extracellular Aβ was associated with higher CAA score. Our data support the feasibility of using the retinal tissue to assess ocular Aβ as a surrogate measure of Aβ in the brain of individuals with AD. Specifically, mid-peripheral retina possesses more Aβ deposition than central retina, and thus may be the optimal location for future *in vivo* ocular imaging.

## Introduction

Dementia is a multifactorial cognitive disorder, impacting memory, performance of daily activities, and communication abilities. It is a major cause of disability and dependency in the world; 47 million people, or 5% of the global geriatric population, are affected, with an estimated annual cost of $818 billion dollars (World Health Organization, 2015). The prevalence and impact establish dementia as a public health and research priority.

Alzheimer’s disease (AD) is the most common form of dementia, characterized by the deposition of amyloid-β (Aβ)-containing senile neuritic plaques and neurofibrillary tangles composed of hyperphosporylated tau in the brain. It accounts for 60–70% of all dementia cases. Although there is no known cure for AD, early diagnosis is important for disease management. AD is known to be underdiagnosed and often recognized only at a later, more advanced stage, and this has been suggested as a contributing factor in the many unsuccessful AD treatments trials (Solomon and Murphy, 2005; Schneider, 2010; Amjad et al., 2018). A definitive diagnosis of AD is possible only by autopsy. Accurate ante-mortem diagnosis is time-consuming and costly, involving cerebrospinal fluid and blood tests along with neuroimaging and neuropsychologic data.

The deposition of Aβ and tau are thought to precede structural imaging abnormalities and cognitive decline by several years (Pike et al., 2007). Aβ in the brain can be detected *in vivo* using positron emission tomography (PET) with Aβ-specific radiotracers such as Pittsburgh compound B (PiB). However, use of PiB-PET is limited by its cost, availability, and exposure to radioactivity. There is a need for preclinical AD biomarkers that are more readily available, less expensive, and less invasive.

In this study, we focus on the retinal manifestation of AD pathology, because the retina is an extension of the central nervous system (CNS) and is uniquely accessible to non-invasive imaging. The retina shares embryonic origin with the brain, as well as similarities in anatomy, function, response to insult, and immunology (London et al., 2013). Aβ has been shown to deposit in the neuroretina, and it is hypothesized that the retina and brain may share basic neuropathological features in AD (Koronyo-Hamaoui et al., 2011; Frost et al., 2014; Hart et al., 2016). Importantly the retina can be readily examined *in vivo* using noninvasive, light-based imaging techniques such as fundus photography and optical coherence tomography (OCT) that are more accessible and cost-effective than neuroimaging. With the potential of a complementary diagnostic tool, multiple groups have begun to investigate the pathophysiology of AD in the retina for visual and ocular biomarkers (Hart et al., 2016; Javaid et al., 2016). Several *in vivo* studies using OCT found reduced retinal layer thickness (Lu et al., 2010; Marziani et al., 2013; Cheung et al., 2015; Coppola et al., 2015) and retinal microvasculature density (Bulut et al., 2018; O’Bryhim et al., 2018) while *ex vivo* studies reported decrease in retinal ganglion cell density and dendritic pruning (Williams et al., 2013).

AD-related Aβ in the retina has been reported in AD-transgenic murine models (Ning et al., 2008; Shimazawa et al., 2008; Alexandrov et al., 2011; Koronyo-Hamaoui et al., 2011; Tsai et al., 2014). However, results from human post-mortem studies have been mixed (Jiang et al., 2016; Shah et al., 2017). Koronyo-Hamaoui et al. (2011); Koronyo et al. (2017), and La Morgia et al. (2016) used immunohistochemistry in wholemount retinas and found increased Aβ plaques in the inner retinal layers of AD donors and suspected early stage AD donors but not in those from normal controls. In contrast, Ho et al. (2014) did not detect amyloid beta deposition after immunostaining on retinal cross sections, a plane of section that minimizes detectability of labeling. den Haan et al. (2018) found no Aβ-related differences between AD and control retina, but did note increased phosphorylated tau in retina without cerebral amyloid angiopathy. Hinton et al. (1986) and Blanks et al. (1989) did not observe amyloid angiopathy using Thioflavin S staining.

Given this variability from multiple groups, this study aims to provide a clearer consensus of the retinal distribution of Aβ in AD donors with a thorough quantitative regional analysis of the retina and correlation with neuropathological assessment. The purpose of our study is to investigate the presence of Aβ in retinal wholemounts from neuropathologically confirmed AD cases, in order to guide future retinal imaging studies targeting Aβ.

## Materials and Methods

### Materials

This study was approved by the Clinical Ethics Research Board of the University of British Columbia and strictly adhered to the Declaration of Helsinki. Human post-mortem brain and retinal tissue from donors with neuropathologically confirmed AD (*n* = 15, mean age = 80.5 ± 8.3 years) were obtained from the Pathology Department at Vancouver General Hospital. All brain samples were evaluated for neuritic and diffuse senile plaques, Aβ protein distribution (Thal phase), neurofibrillary tangle distribution (Braak stage) and cerebral amyloid angiopathy (CAA). Cases with moderate or severe AD with or without other neurological comorbidities were included in the study. Table 1 shows the demographic information and neuropathological assessments.

**TABLE 1:** Demographics, diagnosis, and Alzheimer’s disease pathology measures of the cases.

Case no.	Age	Sex	Primary Path Dx	Additional Path Dx	A-beta (Thal, Biel) (1–5)	Braak stage (1–6)	Neuritic Plaque (CERAD, Biel)	Diffuse Plaque (CERAD, Biel)
1	75	M	N/A	–	–	–	–	–
2	70	M	N/A	–	–	–	–	–
3	75	M	N/A	–	–	–	–	–
4	73	M	N/A	–	–	–	–	–
5	75	M	N/A	–	–	–	–	–
6	66	M	N/A	–	–	–	–	–
7	69	M	N/A	–	–	–	–	–
8	69	M	N/A	–	–	–	–	–
9	55	F	N/A	–	–	–	–	–
10	63	F	N/A	–	–	–	–	–
11	78	F	AD	–	5	6	Frequent	Frequent
12	88	F	AD	FTLD-TDP, CAA	5	6	Frequent	Frequent
13	59	F	AD	DLB	5	6	Frequent	Frequent
14	70	M	AD	CAA	5	6	Moderate	Frequent
15	80	M	DLB	mod. AD	3	4	Moderate	Frequent
16	94	F	AD	CAA	5	6	Frequent	Frequent
17	75	M	AD	DLB, CAA	5	6	Frequent	Sparse
18	82	F	AD	DLB	5	6	Frequent	Frequent
19	82	M	DLB	mod. AD	5	5	Moderate	Frequent
20	79	M	AD		5	6	Frequent	Frequent
21	87	F	CVD	mod. AD	5	4	Moderate	Frequent
22	82	F	AD	CAA	5	6	Frequent	Frequent
23	81	F	AD	mild DLB	5	6	Frequent	Frequent
24	89	F	CVD	mod. AD, FTLD-TDP	5	5	Moderate	Frequent
25	82	F	AD	HS, CAA	5	6	Frequent	Frequent

*AD, Alzheimer’s disease; DLB, dementia with Lewy bodies; CVD, cerebrovascular dementia; FTLD-TDP, frontotemporal lobar degeneration with TDP-43 inclusions; CAA, cerebral amyloid angiopathy; HS, Huntington’s disease; N/A, not applicable (Control eyes).*

Control postmortem eyes (*n* = 10, mean age = 69 ± 6.4 years) were obtained from the Eye Bank of British Columbia. There was a significant age difference between the control and AD (two-sample *t-*test, *p* < 0.001). The control eyes were screened by extensive criteria; those eyes with CNS disorders such as Alzheimer’s disease, Multiple Sclerosis, Parkinson’s disease, and Amyotrophic Lateral Sclerosis were excluded. All tissues were fixed in 10% formalin.

### Retinal Tissue Preparation

**FIGURE 1:**
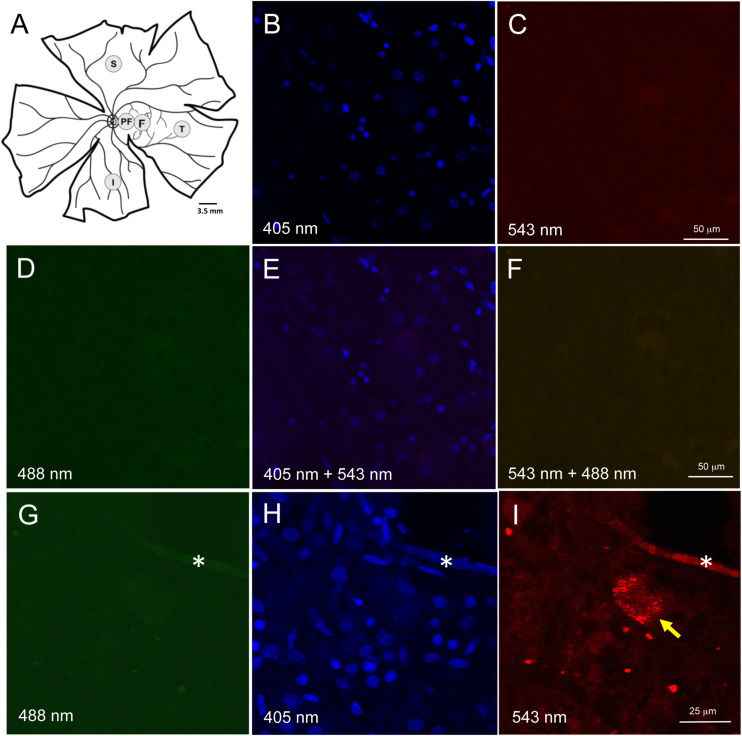
Retinal wholemount tissue processing and controls for autofluorescence. **(A)** Representative drawing of a left wholemount human retina. Punches (3.5 mm diameter) were taken from foveal **(F)** and the peri-foveal (PF) regions. The PF punch was taken midway between the foveal punch and the optic nerve head. Additional punches were also taken from superior (S), temporal (T), and inferior **(I)** quadrants in the mid-peripheral retina. Each mid-peripheral punch was taken approximately 4 disc-diameters from the optic nerve head. **(B–F)** Negative control section of neuroretinal wholemount with DAPI-only staining. Confocal microscope imaging at wavelength 405 nm **(B)**, 543 nm **(C)**, 488 nm **(D)**, 405 and 543 nm **(E)**, 543 and 488 nm **(F)**. There was very low background fluorescence. No autofluorescence was visible. **(G–I)** Neuroretinal wholemount from an AD eye processed for immunofluorescence with anti-Aβ antibody (clone 6F/3D) and imaged at wavelengths 488 nm **(G)**, 405 nm **(H)** and 563 nm **(I)**. The immunofluorescence seen in panel **(I)** is specific for Aβ, as the secondary antibody was labeled with Cy3. Under excitation wavelengths of 405 nm **(H)** and 488 nm **(G)** the immunofluorescent cell seen in panel **(I)** does not display an autofluorescent signal from lipofuscin or melanopsin. Asterisk (*) in **(G–I)** indicates a landmark blood vessel.

The posterior eyecup was prepared by first removing cornea, lens, and iris by careful dissection. Next, the vitreous fluid was removed. The neuroretinal tissue was separated from the retinal pigment epithelium (RPE)/choroid. Free-floating 3.5 mm punches of the neuroretinal layers were taken from the fovea, peri-fovea, and three cardinal directions (temporal, superior and inferior) (Figure 1A). The nasal quadrant was not present in all eye samples due to autopsy procedures and omitted from the study. Multiple punches were obtained from most quadrants for immunohistochemical detection by different Aβ antibodies. The neuroretinal samples were processed by the following procedures. After each step, tissues were washed in phosphate buffered solution (PBS, pH 7.4). First, vitreous remnants were liquefied in 0.07 mg/ml hyaluronidase solution (Sigma, St. Louis, MO, United States). Any remaining vitreous was carefully removed with forceps under a dissecting microscope.

Given AD brain tissue demonstrates lipofuscin autofluorescence, we first assessed the neuroretinal punches for endogenous fluorescent signals which would, if present, require histological steps for its suppression (Dowson and Harris, 1981; Delori et al., 1995). Human retina is known to have lipofuscin accumulation with age, but this is only known to occur in the retinal pigment epithelial cells, and not in the neuroretinal layers. To confirm that lipofuscin did not exist in the neuroretinal tissues, control punches were stained with DAPI 1:500 in PBS for 10 min at room temperature (RT), coverslipped and imaged at 488 nm (green), 563 nm (red) and 406 nm (blue) (Figures 1B–F). Control punches prepared for DAPI staining and imaged at 488 and 563 nm were devoid of autofluorescence under both channels, thus confirming the literature that lipofuscin was not present in the neuroretinal tissues.

A subpopulation of melanopsin-containing retinal ganglion cells (mRGCs) also display autofluorescences under 490 nm excitation (Solomon et al., 1996). To further control against the potential autofluorescence from mRGCs, we used secondary antibodies tagged with Cy3, a fluoroprobe that is excited at a longer wavelength (563 nm), and outside the range of autofluorescence known for melanopsin. As stated above, no autofluorescence was observed in control punches at 563 or 488 nm excitation. Wholemount punches from AD eyes were subjected to Aβ immunohistochemistry and routinely imaged under multiple laser channels including 488, 405, and 563 nm (Figures 1G–I). Fluorescence labeling for Aβ was evident under 563 nm excitation (as expected using the Cy3 tagged secondary antibodies) but fluorescence was not observed under 488 nm, indicating lack of an autofluorescent signal associated with lipofuscin or melanopsin.

**FIGURE 2:**
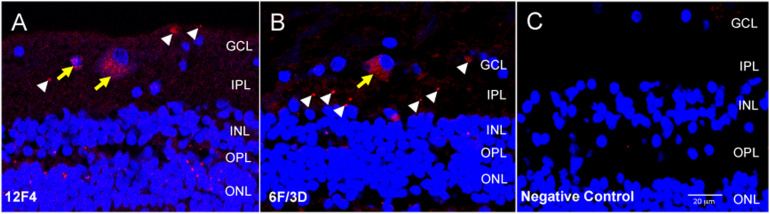
Immunofluorescence with anti-Aβ antibodies 12F4 and 6F/3D in retinal cross sections demonstrated similar labeling patterns. **(A,B)** The immunofluorescence resulting from monoclonal antibodies 12F4 **(A)** and 6F/3D **(B)** was similar and comprised both intracellular (yellow arrows) and extracellular (white arrowheads) labeling. 12F4 recognizes the C’-terminus of the Aβ and is specific for the isoform ending at the 42nd amino acid, while 6F/3D) is specific for Aβ fragments spanning amino acid residues 8–17 of the Aβ peptide chain. **(C)** Replacement of the primary antibody with non-specific mouse IgG2 kappa (for 6F/3D) or IgG1 (for 12F4) isotype antibody yielded no immunoreactivity under Cy3 wavelength, 563 nm.

For immunofluorescence, free-floating punches underwent antigen retrieval with 88% formic acid for 5 min at RT. Punches were also blocked with 3% normal goat serum diluted in 0.3% Triton X (TX)-100-PBS solution for 20 min at RT to minimize non-specific staining. Brain and retinal immunohistochemistry were undertaken with two monoclonal antibodies against Aβ. Clone 6F/3D (Dako, Denmark) is specific for Aβ fragments spanning amino acid residues 8–17 of the Aβ peptide chain (Solomon et al., 1996). 12F4 (Biolegend, San Diego, CA, United States) is specific to the C’-terminus of the Aβ and is specific for the isoform ending at the 42nd amino acid (Mastrangelo and Bowers, 2008). For retinal punches, both 6F/3D and 12F4 antibodies were diluted at 1:100 in normal goat serum and PBS with 3% TX-100 for 1 h at RT, followed by incubation overnight at 4°C. The punches then underwent secondary antibody incubation in goat anti-mouse Cy3 (Jackson Immunoresearch Laboratories Inc., West Grove, PA, United States) diluted at 1:400 in PBS for 45 min at RT. Following antibody incubation, nuclei were stained with DAPI 1:500 in PBS for 10 min at RT. Samples were then slide mounted in glycerol:PBS with No 1.5 coverslip glass. The pattern of immunofluorescence resulting from 12F4 and 6F/3D was very similar and comprised both intracellular and extracellular labeling (Figures 2A,B).

Negative control sections were obtained by incubation with non-specific mouse IgG2 kappa (for BA4) or IgG1 (for 12F4) isotype antibody. This isotype antibody was prepared by diluting 10 μl in 1 ml of PBS to a final concentration of 2.5 μg/ml. All other steps of the protocol were identical. The negative control sections were assessed for non-specific immunoreactivity associated with incubation in the fluorescent secondary antibody. An example of the negative control fluorescent signal is shown in Figure 2C.

### Imaging

**FIGURE 3:**
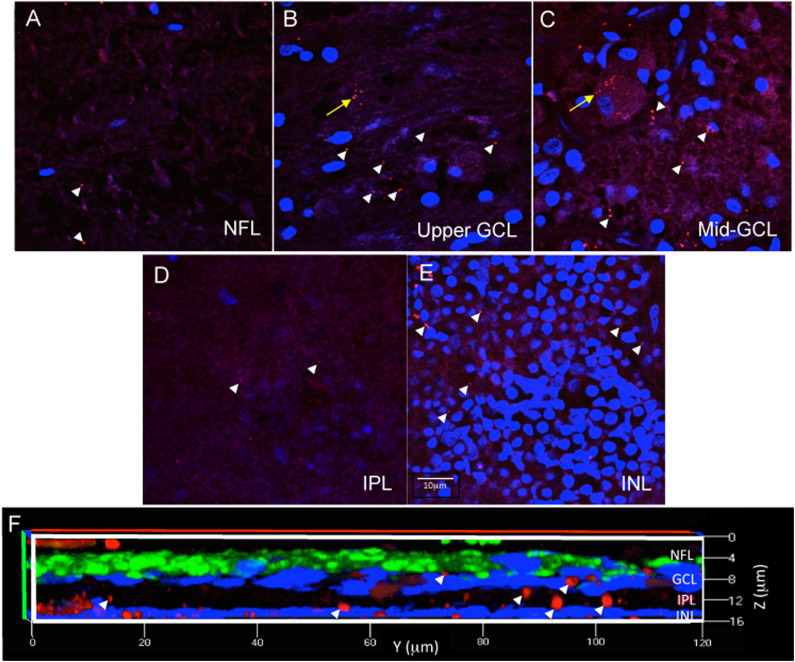
Immunofluorescence in wholemount retina demonstrates antibody penetration into the NFL, GCL, IPL and INL. **(A)** Confocal imaging allows optical sectioning through retina layers and demonstrates that the immunofluorescence was evident in the most superficial retinal layer, the NFL, which is a cell sparse layer as seen by the low number of DAPI labeled nuclei. **(B)** Optical sectioning further into the wholemount reveals the upper part of the GCL, with more DAPI labeled nuclei, more numerous extracellular Aβ deposits (white arrowheads) and the beginning of an RGC with intracellular Aβ labeling (yellow arrow). **(C)** Continuation of the same RGC is shown more completely labeled in the middle of the GCL. **(D)** Progressing deeper into the wholemount, the IPL, another cell sparse layer, is shown with minimal extracellular Aβ immunoreactivity. **(E)** Lastly, the INL is identified by the high density of DAPI labeled nuclei, which are generally smaller in diameter than observed for the nuclei of RGC. There are extracellular deposits, but few INL neurons demonstrating Aβ intracellular labeling. Scale bar for panels **(A–E)** = 10 μm. **(F)** A magnified, orthogonal view of a set of images from an AD eye wholemount Z-stack demonstrates Cy3 (red) Aβ immunofluorescence (arrows) in the NFL, GCL, IPL, and INL. The green fluorescence is beta-3 tubulin immunoreactivity demonstrating neuronal processes associated with retinal ganglion cell axons in the NFL. Abbreviations: NFL, nerve fiber layer; GCL, ganglion cell layer; IPL, inner plexiform layer; INL, inner nuclear layer.

For each eye, free-floating punches from five regions (fovea, peri-fovea, superior, temporal, inferior) were imaged using a Zeiss 510 confocal microscope with LSM510 or Zen 2009 software focusing at the level of the retinal ganglion cells (RGC). The retinal ganglion cell layer was identified by sequentially imaging through the thickness of the punch, starting from the nerve fiber layer (NFL, a cell free layer), then proceeding to the ganglion cell layer (GCL, in which DAPI labeled nuclei are abundant), then further into the inner plexiform layer (IPL, a cell free layer) and finally into the inner nuclear layer (INL, in which DAPI labeled nuclei are abundant) (Figures 3A–E). The series of z-stack images confirmed that the antibody incubations penetrated into the punches, as labeling was observed as deep as the INL in orthogonal reconstructions of z-stack images (Figure 3F). As this study addresses Aβ labeling principally in the GCL, the methods described above were deemed appropriately optimized for immunofluorescence in human wholemount, retinal punch tissues.

Cy3 immunofluorescence from both the 12F4 and 6F/3D antibodies was imaged at 543 nm and nuclear labeling at 405 nm at 20×, 40× and 63× magnifications. Each punch was imaged in four non-overlapping regions. When choosing the four regions to image, care was taken to avoid regions with artifactual tears in tissues and areas obscured by large retinal vessels. Each imaging field view of 450 μm^2^ × 450 μm^2^ was collected. The total imaging field view spanned approximately 50% of the accessible punch tissues.

### Aβ Measurement

Trained evaluators loaded each confocal image into NIH ImageJ (Schneider et al., 2012) and delineated artifacts and blood vessels to exclude. The evaluators were masked to the group status (control or AD). In the remaining area, Cy3 labeling was categorized into two groups: intracellular labeling inside the cell bodies, identified as distinct clusters of immunoreactive dust-like deposits within the cytoplasmic compartment surrounding a DAPI labeled nuclei, and extracellular labeling, identified as denser dot-like staining within the neuropil (Figures 2, 3). All visible Cy3-labeled cell bodies were manually delineated. This divided the image into three non-overlapping regions: (i) artifact/vessel, (ii) intracellular region, and (iii) the rest of the image, denoted as the extracellular region.

Aβ load in each image was assessed using the following quantitative measures: (i) number of Cy3-labeled cells (per image area of 450 μm^2^ × 450 μm^2^), and (ii) percentages of Cy3-labeled pixels in the intracellular and extracellular regions. Intracellular Aβ load was defined as the percentage of Cy3-positive pixels in the intracellular region, and the extracellular Aβ load was defined as the percentage of Cy3-positive pixels in the extracellular region.

For each punch, the parameter values were averaged over the four quadrant images. All image processing was performed using MATLAB R2016a (The Mathworks, Inc).

### Statistics

The number of labeled cells and pixel-wise intracellular and extracellular Aβ loads were compared between the diagnosis groups (Control, AD) using a non-parametric, robust ANCOVA (Kloke et al., 2014) with age as a covariate. The parameter values were compared also across the retinal regions within each group, and within the AD group by neuropathological assessments using two-samples Wilcoxon test. The results were plotted in a scatter box plot for each group. In the box plot, the top and bottom of the box are the third and first quartiles [Q3 and Q1, interquartile range (IQR)], and the bold line inside the box is the median. The top and bottom whisker ends are the highest data point within Q3 + 1.5 × IQR and the lowest data within Q1 – 1.5 × IQR, respectively. Each parameter was also plotted against the subject age, and the correlation between subject age and retinal Aβ load was tested using Spearman’s rank correlation coefficients (Gauthier, 2001; van Belle et al., 2004). The statistical analysis and visualization were performed using R (R Core Team, 2019).

## Results

### Comparison of Retinal Aβ Immunofluorescence Between Control and AD

**FIGURE 4:**
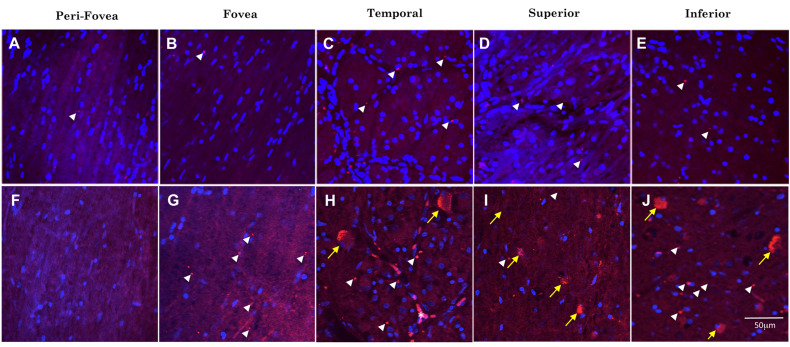
Comparison of Aβ immunofluorescence in the AD and control groups. **(A–E)** Confocal images, taken at the level of the GCL, of the perifoveal, fovea, temporal, superior and inferior regions of the retina from a control wholemount. **(F–J)** Confocal images taken from an AD wholemount for comparison. There were generally higher levels of Aβ immunofluorescence (Cy3, red) in the AD compared to the control groups. In addition, there are fewer DAPI labeled nuclei in the AD compared to control groups, consistent with the earlier studies demonstrating neurodegeneration and loss of retinal ganglion cells in the AD eye. The mid-peripheral areas (temporal, superior and inferior) demonstrated more Aβ labeling than the central areas (perifovea and fovea) of the retina. The pattern of Aβ immunofluorescence consisted of strongly immunoreactive, dot-like extracellular neuropil deposits (white arrowheads) and dust-like cytoplasmic labeling within RGCs (yellow arrows). An occasional blood vessel lumen was also labeled (**H**, asterisk), but the labeling was dull and opaque, quite distinct from the bright, dot-like immunofluorescence seen in the extracellular deposits or the bright, dust-like cytoplasmic labeling. Nuclei are shown stained with DAPI (blue). Scale bar = 50 μm.

Figure 4 shows representative images of Aβ immunofluorescence in 5 regions of the wholemount retina in controls (Figures 4A–E) and AD (Figures 4F–J). Note that the analysis was undertaken at the level of the retinal ganglion cell layer (GCL), as verified by the presence of numerous DAPI-labeled nuclei in Z-stack confocal files (Figure 3). The mid-peripheral areas (temporal, superior and inferior) demonstrated more Aβ labeling than the central areas (perifovea and fovea) of the retina. The pattern of Aβ immunofluorescence in the GCL consisted of strongly immunoreactive, dot-like extracellular neuropil deposits and dust-like cytoplasmic labeling within RGCs. An occasional blood vessel lumen was also labeled (Figure 4H, asterisk), but the labeling was dull and opaque, quite distinct from the bright, dot-like immunofluorescence seen in the extracellular deposits or the bright, dust-like cytoplasmic labeling. Fluorescence from the retinal vasculature was not included in the analysis here. Note that upon visual comparison between controls (Figures 4A–E) and AD (Figures 4F–J), it is apparent that the AD wholemounts had higher levels of Aβ immunofluorescence compared to controls. This qualitative difference was then subjected to quantitative analysis in section “Quantitative Analysis of Retinal Aβ Between Control and AD.” In addition, the AD wholemounts demonstrated fewer DAPI labeled nuclei when compared to the controls, consistent with the earlier findings that the ganglion cells undergo neurodegeneration in the AD eye (Blanks et al., 1996a, b).

**FIGURE 5:**
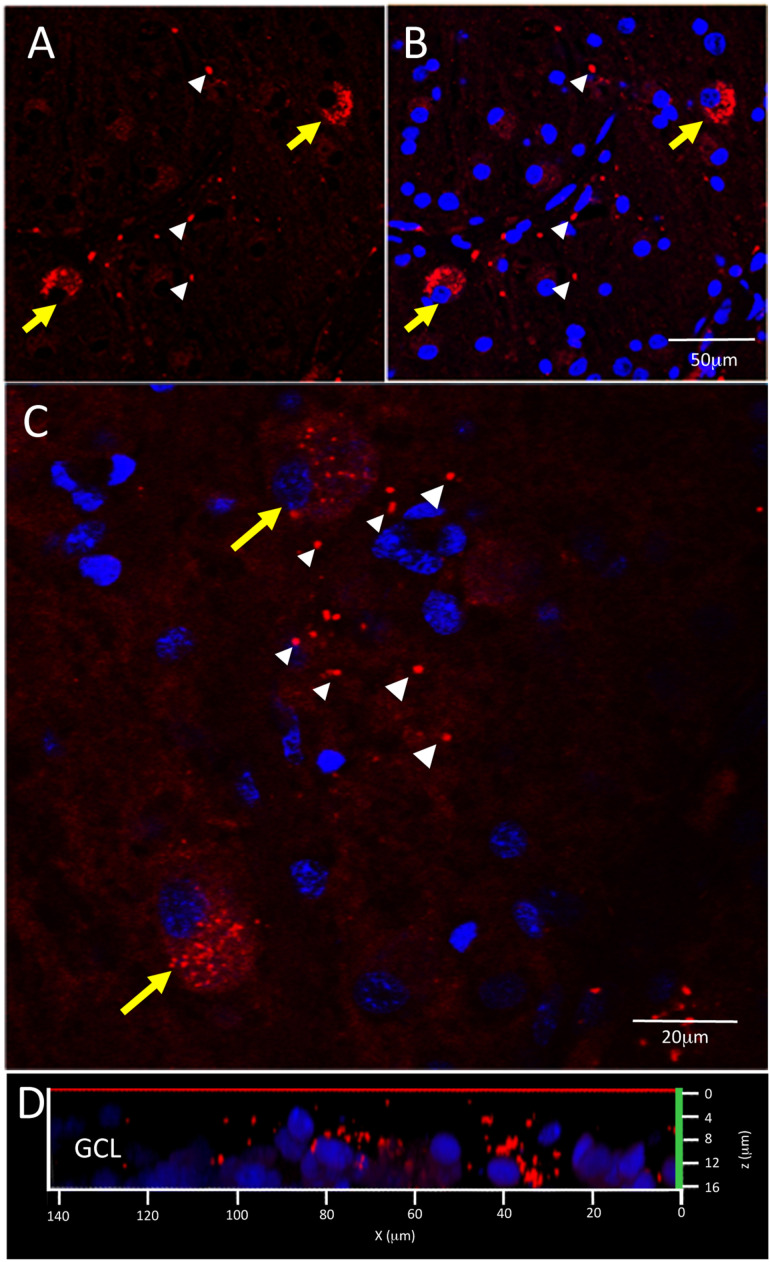
Control and AD retina demonstrate similar pattern of intracellular and extracellular Aβ immunofluorescence. **(A,B)** Confocal image of a control retina demonstrates intracellular Aβ labeling (yellow arrows) which consisted of small, dust-like cytoplasmic deposits. **(C)** A higher power confocal image of an AD retina reveals the same pattern of intracellular labeling as observed in the control retina and also demonstrates extracellular Aβ labeling (white arrowheads) composed of relatively larger dot-like deposits compare to the intracellular labeling. **(D)** An orthogonal view of the z-stack demonstrates the Aβ labeling in the GCL. Scale bar = 50 μm (A, B) or 20 μm **(C)**.

Figure 5 shows examples of labeling patterns in control (Figures 5A,B) and AD (Figures 5C,D) groups. Note that there is a similar pattern of bright, dot-like extracellular deposits and bright, dust-like cytoplasmic labeling in both the AD and control groups.

### Quantitative Analysis of Retinal Aβ Between Control and AD

**FIGURE 6:**
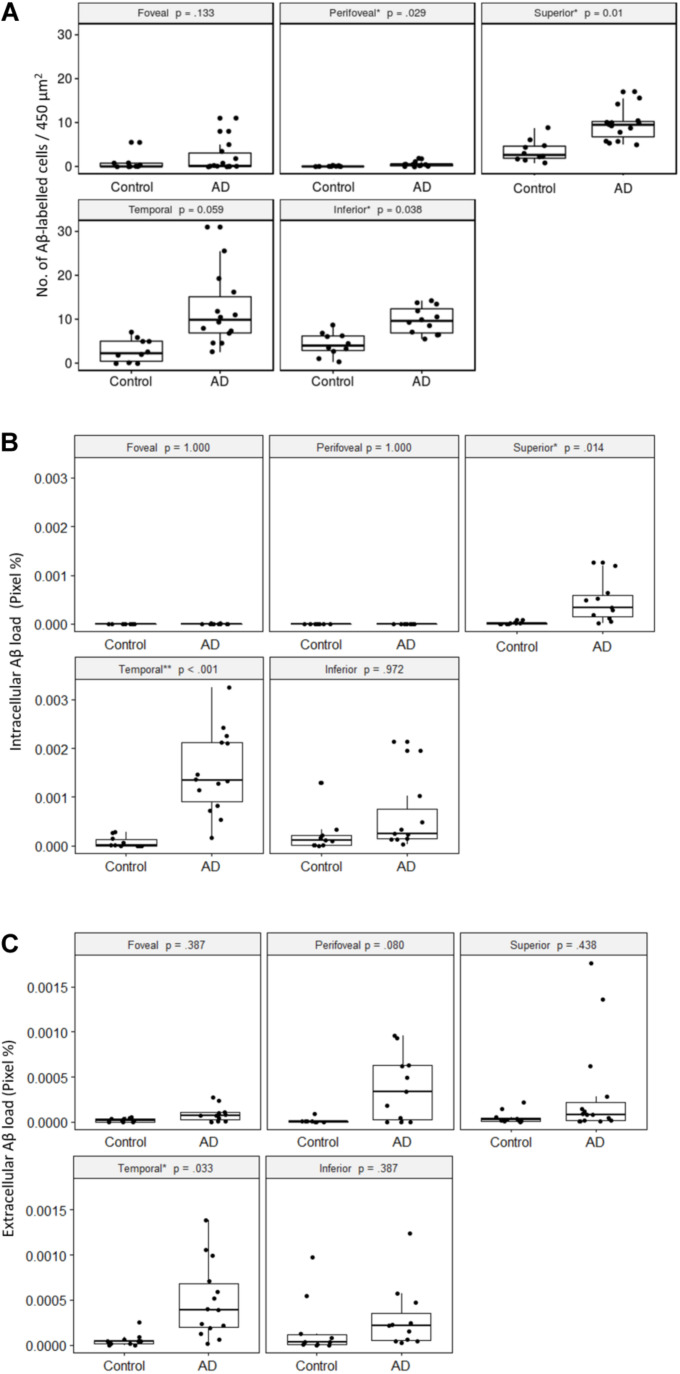
Comparisons of Aβ-labeling between control and AD groups. **(A)** Number of Aβ-labeled cells in the retinal ganglion cell layer. The AD subjects had significantly greater numbers of Aβ-labeled cells than the controls in the peri-foveal, superior, and inferior regions, using age-adjusted robust ANCOVA. **(B)** Intracellular Aβ load in the retinal ganglion cell layer. The AD subjects had significantly greater intracellular Aβ load than the controls in the superior and temporal regions using age-adjusted robust ANCOVA. **(C)** Extracellular Aβ load in the retinal ganglion cell layer. The AD subjects had significantly greater extracellular Aβ load than the control in the temporal region using age-adjusted robust ANCOVA.

First, we quantified the number of Aβ labeled cells in the GCL per image area of 450 μm^2^ × 450 μm^2^ in the control and AD subjects in each retinal region, with the *p*-values from robust ANCOVA adjusting for age (Figure 6A). The AD group had significantly more Aβ labeled cells than the control group in the peri-foveal, superior, and inferior regions. Age was not a significant covariate in any region.

Within both control and AD groups, there were significantly more Aβ labeled cells (*p* < 0.05) in the mid-peripheral regions (superior, temporal, inferior) than the central regions (foveal, perifoveal).

Next, we quantified the percentage of Cy3-positive pixels within each intracellularly labeled cell (e.g., intracellular Aβ load) in the control and AD subjects for each retinal region (Figure 6B). The foveal and perifoveal regions contained only a few labeled cells and the intracellular Aβ load is close to zero in both control and AD groups. However, amongst the mid-peripheral regions, there was a general trend for the AD group to display higher levels of intracellular Aβ load compared to controls. This was statistically significant in the superior (*p* = 0.01) and temporal (*p* < 0.001) retinal regions. Age was not a significant covariate in any region.

Within the control group, there was significantly greater intracellular Aβ load (*p* < 0.05) in the mid-peripheral regions than the perifoveal region. The differences between the mid-peripheral regions and foveal region were not significant except for the inferior region (*p* < 0.01).

Within the AD group, the difference between the central (foveal, perifoveal) and mid-peripheral regions was significant and more pronounced than in the control group. Amongst the mid-peripheral regions, the temporal region in AD had significantly greater intracellular Aβ load than the superior region in AD (*p* < 0.05). There was no significant difference between the foveal and perifoveal regions.

We then quantified the extracellular Aβ load in the control and AD groups in each retinal region (Figure 6C). The AD group had significantly greater extracellular Aβ load in the temporal region compared to controls. Age was not a significant covariate in any region.

Compared to the intracellular Aβ measures, extracellular Aβ load was more evenly distributed across the retinal regions. In the control group there was no significant regional difference. However, in the AD group, the extracellular Aβ load was significantly higher in the temporal region than in the superior region (*p* = 0.05). Overall, in the AD group the temporal region showed greater amount of both intracellular and extracellular Aβ load compared to other retinal regions.

### Comparison Within AD Subjects

We obtained neuropathological assessments of the AD subjects and compared with retinal measurements of Aβ. Supplementary Figure 4 plots the neuropathological assessments of the AD subjects. The retinal measurements, as shown above in Figure 6, were continuous variables with intra-group variances; however, the neuropathological scores were discrete categories based on severity, and did not have similar spreads as the retinal measurements which made comparisons difficult. For example, cerebral Aβ deposits, 1 out of 15 AD subjects was in Thal Phase 3, while the rest were in Phase 6 (Supplementary Figure 4A). For neurofibrillary degeneration, 2 subjects each were in Braak Stages IV and V, and 11 subjects were in Stage VI (Supplementary Figure 4B). However, two neuropathological assessments showed some spread among the AD subjects that allowed for comparison of the retina Aβ by their subgroups: CERAD scores for neuritic plaques (NP) and cerebral amyloid angiopathy (CAA) (Supplementary Figures 4D–F,I). Since Aβ deposition in the AD retinas was sparse in the central regions and not significantly greater than the control retinas, we only included the superior, inferior, and temporal regions in the following analysis.

### Comparison of Retinal Aβ Measures by NP and CAA

**FIGURE 7:**
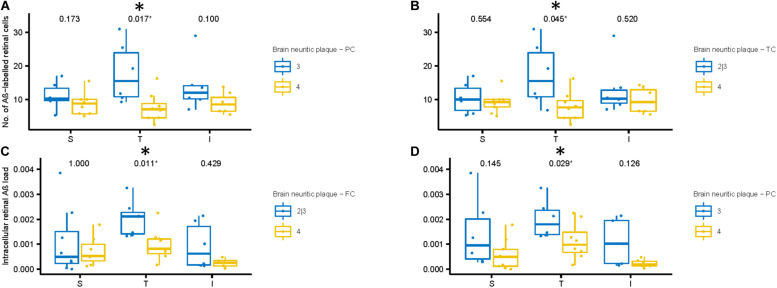
Aβ labeling in retina compared to neuritic plaque (NP) scores in cortical regions of AD subjects. **(A,B)** We compared number of Aβ labeled cells with NP scores. The AD subjects with lower NP scores (Blue: sparse or moderate) in the parietal **(A)** and temporal **(B)** cortices show greater number of Aβ labeled cells in the temporal retinal region than those with higher NP scores (Yellow: frequent), using two-samples Wilcoxon test. **(C,D)** Next, we compared intracellular Aβ load in the retinal ganglion cells with NP scores. The AD subjects with lower NP scores (Blue: sparse or moderate) in the frontal **(C)** and parietal **(D)** cortices show greater intracellular Aβ load in the temporal retinal region than those with higher NP scores (frequent), using two-samples Wilcoxon test.

Figures 7A,B shows the number of Aβ labeled cells in the AD subjects plotted by NP scores of sparse/moderate (Blue = Lower NP score) and frequent (Yellow = Higher NP score), with *p*-values of unpaired two-samples Wilcoxon test. Note we observed a trend for AD subjects with lower NP scores exhibiting greater numbers of Aβ labeled cells in the mid-peripheral retina, and this reached significance in the temporal retinal region (*p <* 0.05).

Next, we assessed the relationship between the intracellular retinal Aβ load and NP scores in the AD subjects (Figures 7C,D). Again, as with number of Aβ labeled cells, we observed a general trend for AD subjects with lower NP scores (Blue) exhibiting greater intracellular retinal Aβ load compared to AD subjects with higher NP scores (Yellow). This reached significance in the temporal retinal region (*p* < 0.05). Lastly, we assessed the relationship between extracellular retinal Aβ load and NP scores, but this did not reach significance (Supplementary Figures 5A–C).

Conversely, extracellular retinal Aβ load did reach significance with measurements of the severity of CAA in AD subjects. The extracellular retinal Aβ load in the temporal mid-peripheral retina was greater in the AD subjects that exhibited higher CAA severity index compared to the less severe CAA group (*p* < 0.05, Supplementary Figure 5D).

### Age and Retinal Aβ Load

In the ANCOVA models, age was not found as a significant covariate, and no significant interaction was found between group and age. However, Spearman’s correlation coefficient was significant (α < 0.05) for intracellular and extracellular retinal Aβ loads in the temporal region for control and AD groups. Supplementary Figures 1–3 plots the number of Aβ-labeled cells, intracellular retinal Aβ load, and extracellular retinal Aβ load against age.

## Discussion

In this study, we measured the Aβ load in the post-mortem retinas from AD and control subjects using immunohistochemistry and quantitative fluorescence analysis. Intracellular and extracellular retinal Aβ loads were measured in wholemount images focused on the retinal ganglion cell layer (GCL) in the foveal, perifoveal, superior, temporal, and inferior retinal quadrants, comparing between the AD and control groups. Subgroups among the AD subjects were further analyzed by their neuropathological assessments, specifically neuritic plaque (NP) scores, and by the degree of cerebral amyloid angiopathy (CAA) (Supplementary Figure 4I).

Our results demonstrated morphologically distinct intracellular and extracellular deposition of retinal Aβ, and both were significantly higher in the retina of AD subjects than controls, mainly in the mid-peripheral retina. Intracellular Aβ measures were significantly greater in the mid-peripheral than the central regions, whereas extracellular Aβ load was more evenly distributed within all retinal regions studied.

Among the AD donors, a significantly higher number of Aβ labeled retinal cells as well as a significantly greater amount of retinal intracellular Aβ were observed in the donors with a lower NP score. In contrast, a significantly greater amount of retinal extracellular Aβ was observed in the AD donors with higher CAA score. Interestingly, the temporal retina demonstrated the most differences related to the brain NP and CAA scores, as well as showing a large difference between the AD and control groups. These findings have important implications for future *in vivo* retinal Aβ imaging studies as the detection and level of retinal Aβ are influenced by factors beyond the severity of AD.

### Intracellular Aβ

Our results on the number and intracellular load of Aβ labeled cells allowed us some insight into the average diameter of the Aβ labeled cells. Our results suggest that the average diameter of Aβ labeled cells was not significantly different between the control and AD groups. This agrees with the results by Blanks, Torigoe, Hinton, and Blanks (Blanks et al., 1996b). Similarly, Padurariu et al. found the hippocampal neuron diameter was not significantly different between the control and AD groups (Padurariu et al., 2012). To better isolate potential differences between AD and controls, future studies may focus on the detailed dendritic morphology of the retinal cells in AD, and the difference between the cells with and without intracellular Aβ labeling.

### Extracellular Aβ

Extracellular Aβ was present in all retinal regions studied in both control and AD subjects. Interestingly, the AD subjects had significantly greater extracellular Aβ load in the temporal retinal region compared to controls (*p* = 0.05). The temporal region of the retina may be of special pathological significance to the AD eye, as our earlier study demonstrated that temporal retina had significantly more intermediate hard drusen in the outer retina (Ukalovic et al., 2018), a finding that is consistent with an earlier report by Lengyl’s group (Csincsik et al., 2018).

### Intra- and Extracellular Aβ Relationship

The relationship between intra- and extracellular Aβ is yet to be fully elucidated. In the AD brain, it has been suggested that Aβ accumulation begins intracellularly, and following degeneration from within the neuron, leads to more extracellular deposition and eventual plaque formation (Gouras et al., 2010). In our study, the AD donors with higher neuritic plaque scores had lower retinal intracellular Aβ, a finding that is consistent with the proposed “intracellular to extracellular” mechanism of cortical Aβ deposition of Gouras et al. (2010).

Interestingly, the AD donors with higher neuritic plaque scores did not demonstrate evidence of higher extracellular Aβ in the retina. This suggests that if the intracellular Aβ causes degeneration of retinal ganglion cells, it does not lead to the same kind of extracellular plaque formation as in the brain, where the remnant of destroyed distal neurites and synapses recruit inflammatory cells to initiate plaque formation (Gouras et al., 2010). Thus, further studies are needed to understand the mechanistic differences of Aβ pathophysiology in the retina and the brain, in terms of how intracellular or extracellular Aβ may contribute to retinal neuronal damage, as well as the clearance mechanisms of intracellular Aβ after retinal neuronal death. Answers to these questions are important to guide the development of retinal AD screening tests, as well as to understand the neurodegenerative consequences of Aβ deposition in the AD retina.

### Temporal Pattern of Aβ Deposition

An important, yet unanswered, question is the detailed time-course of Aβ deposition and AD progression, and whether Aβ accumulates in the retina and CNS concurrently in AD patients This is difficult to ascertain from postmortem tissues as the majority of available samples are from late stage disease, thus providing only a static snapshot of the disease progression. Using the APP/PS1 transgenic AD model, Sidiqi et al. (2020) reported an age-related and concomitant increase in Aβ deposition in the cerebral cortex and retina of mice by following mice between the ages of 5–18 months. Another study suggested that retinal Aβ deposition precedes cerebral cortical Aβ deposition (Koronyo-Hamaoui et al., 2011). Other transgenic AD mouse studies have shown a general age-related increase in retinal Aβ deposits (Ning et al., 2008; Perez et al., 2009; Hoh Kam et al., 2010; Gupta et al., 2016; Hart et al., 2016) a finding that also occurs in non-AD (control) mouse and rat strains as well (Hoh Kam et al., 2010; Zhao et al., 2015). In the present study, donor age was positively correlated with intracellular and extracellular Aβ in the temporal region for both control and AD. Although age was not a significant covariate in the ANCOVA models, we also found no significant difference in the effect of age between the control and AD groups. A larger study would be needed to study the age-related deposition of retinal Aβ in human donors and the interaction between the effects of age and AD.

### Comparisons With Previous Studies

Several studies have addressed Aβ deposition in the AD eye, with multiple groups reporting negative findings (Shah et al., 2017). Our results do not support the literature reporting negative findings. Rather, our results do support Aβ deposition in the AD eye. We observed similarities with studies by Koronyo et al. (2017). We found Aβ labeling predominantly in the inner retina (specifically the RGC layer), presence of intracellular Aβ labeling and greater immunoreactivity in mid-peripheral retina compared to the central (foveal, perifoveal) retina. Congruent with findings from the Koronyo-Hamaoui lab, our results also showed significant differences between the normal and AD eyes in both superior and temporal retinal quadrants for intracellular Aβ and the temporal retinal quadrant for extracellular Aβ.

Although the majority of our findings were consistent with the literature, our studies did differ in the size of the extracellular Aβ deposits in the retina. We observed small dot-like extracellular deposits with diameters less than 5 μm, which is in contrast to the findings of Koronyo et al. (2017) and La Morgia et al. (2016) who identified extracellular deposits that were >20 μm in diameter and were similar to the diffuse, compact and “classical” mature plaques observed in AD brain. This does not seem to be related to the antibodies used, as both labs used 12F4, among others (e.g., 6F/3D). The observed differences between the extracellular labeling patterns may be associated with differences in the source of human diseased and control eye tissues (e.g., human tissue banks), the duration of fixation times (time between death, enucleation, fixation and processing), and methods of visualization and imaging (chromogenic vs fluorescent).

### AD-Related Vascular Changes in the Retina

The extracellular retinal Aβ association with severe CAA observed in our current study may indicate its potential role in vascular decline in AD retinas. In a previous study, Aβ deposits immunoreacted with the 12F4 monoclonal antibody were found adjacent to and within blood vessel walls in retinal wholemounts from AD patients with CAA (Koronyo et al., 2017). In *in vivo* studies using retinal fundus photography, narrower retinal venules and sparser retinal vasculature were observed in AD patients as compared to non-dementia controls (Cheung et al., 2014; Williams et al., 2015). A more recent study using Optical Coherence Tomography Angiography (OCTA) found significantly lower retinal vascular density, larger foveal avascular zone, and thinner choroid in AD patients compared to age- and sex-matched controls (Bulut et al., 2018).

### Advantages and Limitations of the Study

The advantages of this study are the comprehensive retinal regional analysis and qualitative and quantitative assessment of intra- and extracellular retinal Aβ load, along with a comprehensive and thorough neuropathological assessment which allowed for comparison between the Aβ load in the retina and neuritic plaque and CAA measures in the brain. The results reveal a connection between the retinal Aβ and AD neuropathology and inform on factors that may affect the utility of potential *in vivo* imaging of Aβ in the retina of AD subjects.

The limitations of this study include the small sample size (control *n* = 10, AD *n* = 15) and the age difference between the controls (average age: 69 ± 6.4 years) and AD (average age: 80.5 ± 8.3 years; two-sample *t-*test *p* < 0.001) donors. All comparisons between the control and AD retina Aβ were adjusted for age, and in neither the non-parametric ANCOVA nor parametric multiple regression showed age as a significant covariate with the group effect (control or AD) present. However, separate correlation analysis showed significant relationship between age and temporal retinal Aβ load. In our small sample AD and CAA pathology had a stronger effect on retinal Aβ accumulation than age. The effect of age may be more subtle, and a more detailed study on normal subjects from different age groups would better characterize this point. CAA can occur as a normal process of aging (Yamada et al., 1987), with 50% of individuals older than 80 years old having some evidence of CAA (Gugenheim et al., 1987). In this study, we did not have CAA severity information on the control brain samples, but the AD patients with no or mild CAA severity still had significantly higher intracellular retinal Aβ load than controls (data not shown). Future investigation involving more specific sample data may further clarify the association of the retinal Aβ with AD and related brain pathology.

### Implications for *in vivo* Imaging of the AD Retina

The results of this study demonstrate that retinal Aβ is present and significantly higher in the AD compared to control retina. These data, along with previous results from the Koronyo-Hamaoui group, highlight the feasibility and potential of *in vivo* imaging of retinal Aβ load in the AD eye. The superior and temporal retina quadrants showed the most significant differences between the control and AD groups. Additionally, we observed variability in the retinal Aβ loads among the AD eyes by their neuritic plaque and CAA severity. Future studies will allow us to further understand the utility of retinal Aβ for diagnostic interpretation and its relationship to neuropathological assessments.

## Data Availability Statement

The raw data supporting the conclusions of this manuscript will be made available by the authors upon appropriate request.

## Ethics Statement

The studies involving human participants were reviewed and approved by University of British Columbia Clinical Research Ethics Committee. The patients/participants provided their written informed consent to participate in this study.

## Author Contributions

SL and JM wrote the manuscript, analyzed and interpreted the data, and generated figures. SL wrote data analysis algorithms for confocal images, completed statistical analysis. JC, ET, QX, and BM processed immunofluorescence on retinal wholemounts and cross sections, captured confocal images and compiled retinal image data. JC dissected and punched eye tissues. ET generated figures, assisted in manuscript preparation and submission. BE and KJ assisted in ophthalmic assessment, figure preparation and provided expert opinion on retinal ganglion cells. IM, VH-R, and G-YH consented AD subjects, obtained neurological assessments, neuropathological analysis of postmortem brain samples and provided expert opinion on pathophysiology of Alzheimer’s disease and other dementias. JM, MS, and MB conceived and designed the study, obtained funding, supervised trainees/staff and critically interpreted the data. All authors read and approved the final draft of the manuscript.

## Conflict of Interest

The authors declare that the research was conducted in the absence of any commercial or financial relationships that could be construed as a potential conflict of interest.

## Supplementary Material

FIGURE S1 Retinal measure of Aβ-labeled cells plotted against subject age.

FIGURE S2 Retinal measure of intracellular Aβ load plotted against subject age.

FIGURE S3 Retinal measure of extracellular Aβ load plotted against subject age.

FIGURE S4 Distribution of neuropathological assessment in the study data. **(A)** Aβ (Thal-phase); **(B)** Braak stage (Tau); **(C)** Neuritic Plaque (CERAD, Biel); **(D)** Neuritic Plaque Frontal Cortex; **(E)** Neuritic Plaque Temporal Cortex; **(F)** Neuritic Plaque Parietal Cortex; **(G)** Diffuse Plaque (CERAD, Biel); **(H)** Likelihood of AD dementia based on the amyloid Thal phase, tau Braak stage, and CERAD score (ABC score); **(I)** Cerebral Amyloid Angiopathy (Congo red + Aβ).

FIGURE S5 Extracellular retinal Aβ load, grouped by **(A)** Neuritic plaque in frontal cortex (sparse (2) or moderate (3), frequent (4)); **(B)** Neuritic plaque in parietal cortex [moderate (3), frequent (4)]; **(C)** Neuritic plaque in temporal cortex [sparse (2) or moderate (3), frequent (4)]; **(D)** cerebral amyloid angiopathy [none (1) or mild (2), moderate (3) or severe (4)].
