# Supplementary figures and images for: Amyloid Beta Immunoreactivity in the Retinal Ganglion Cell Layer of the Alzheimer’s Eye
Source: Front Neurosci. 2020 Jul 31;14:758. doi: 10.3389/fnins.2020.00758 (PMC7412634; doi:10.3389/fnins.2020.00758)

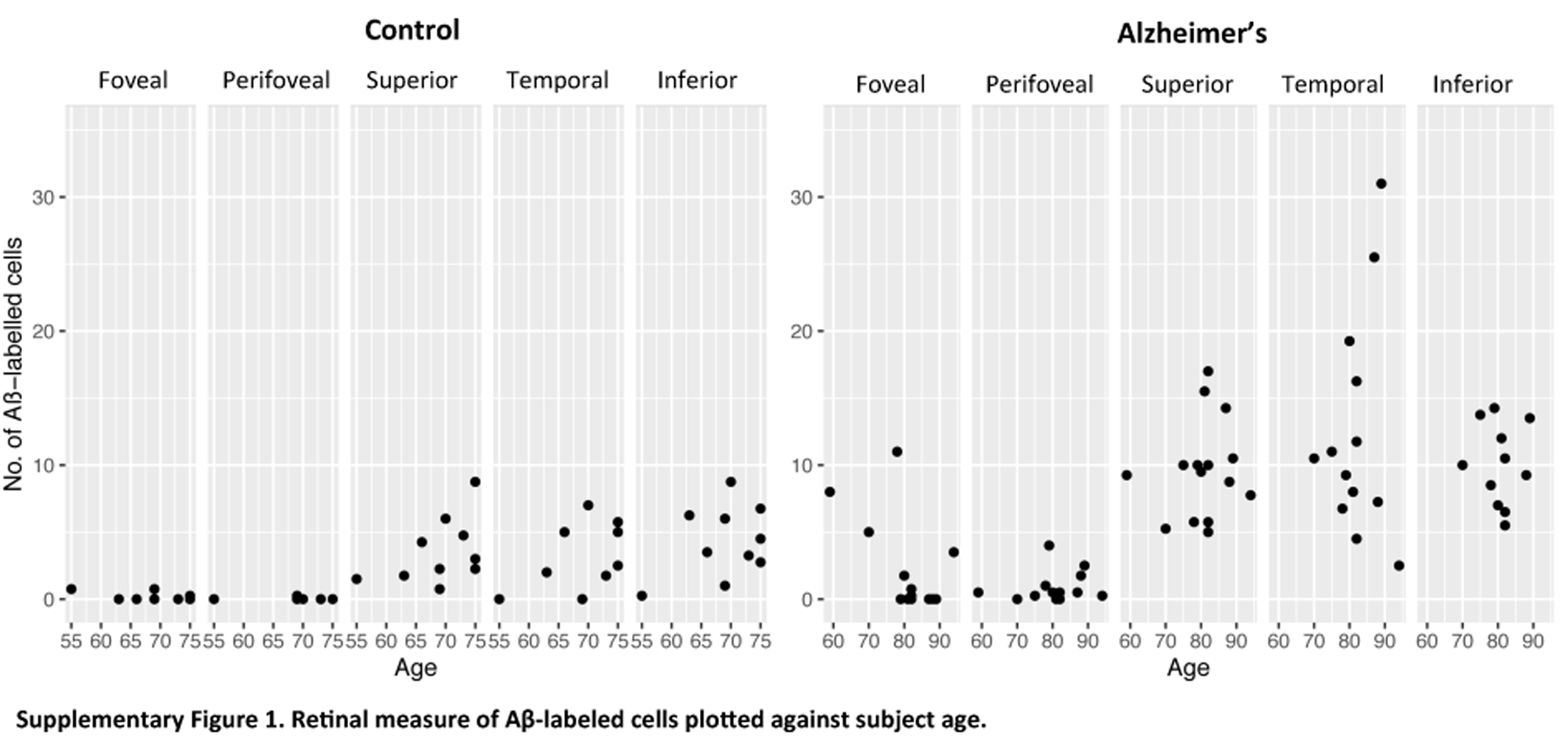

Supplement: FIGURE S1 — Retinal measure of Aβ-labeled cells plotted against subject age. [file Image_1.TIF]

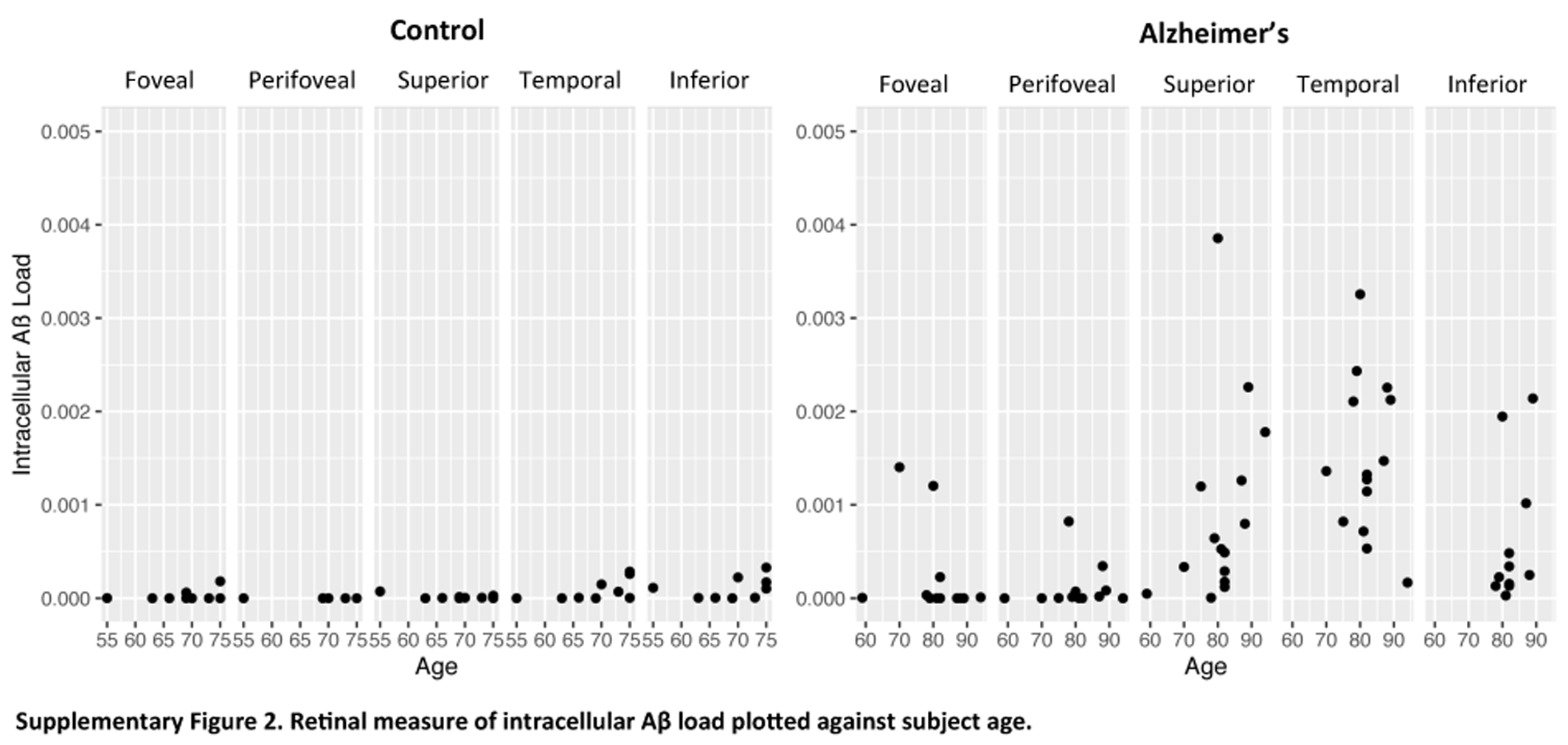

Supplement: FIGURE S2 — Retinal measure of intracellular Aβ load plotted against subject age. [file Image_2.TIF]

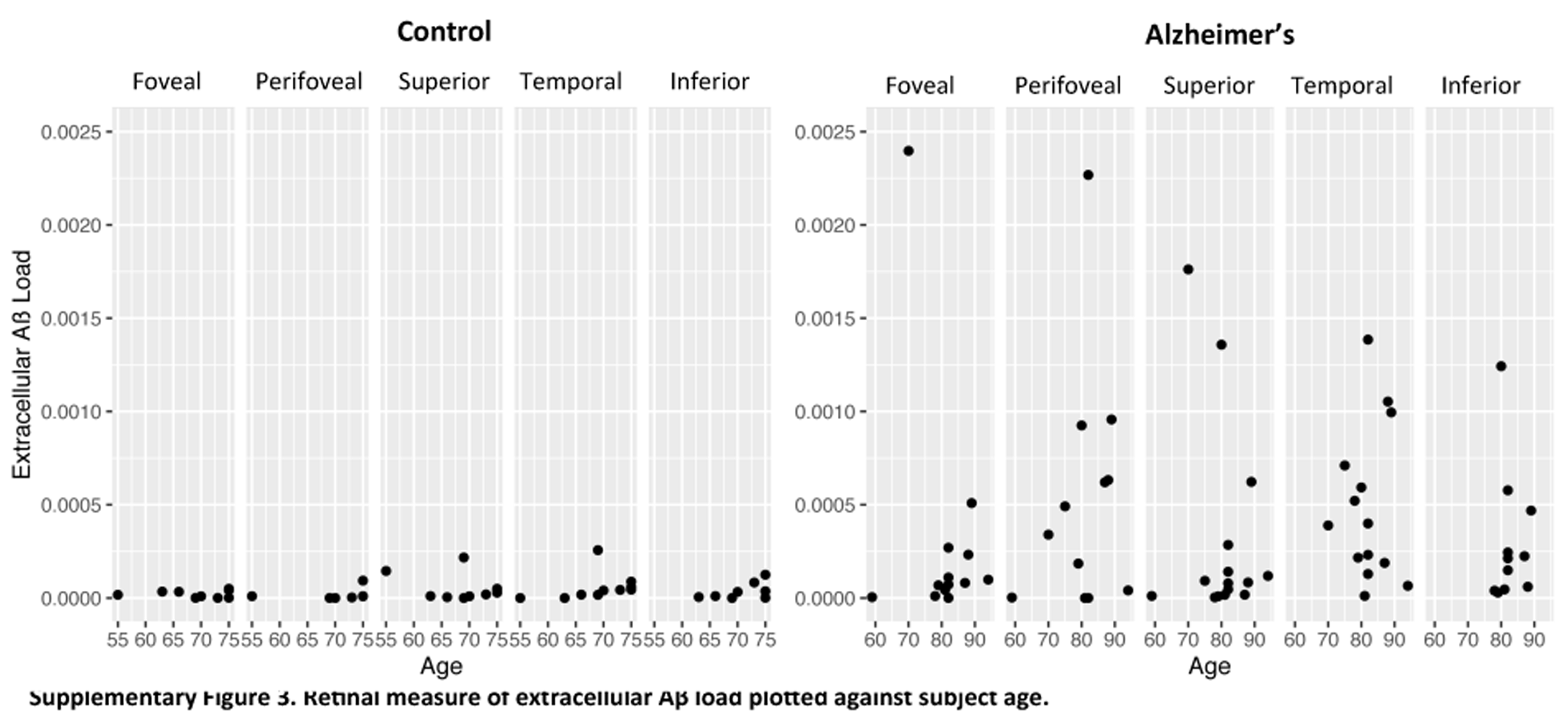

Supplement: FIGURE S3 — Retinal measure of extracellular Aβ load plotted against subject age. [file Image_3.TIF]

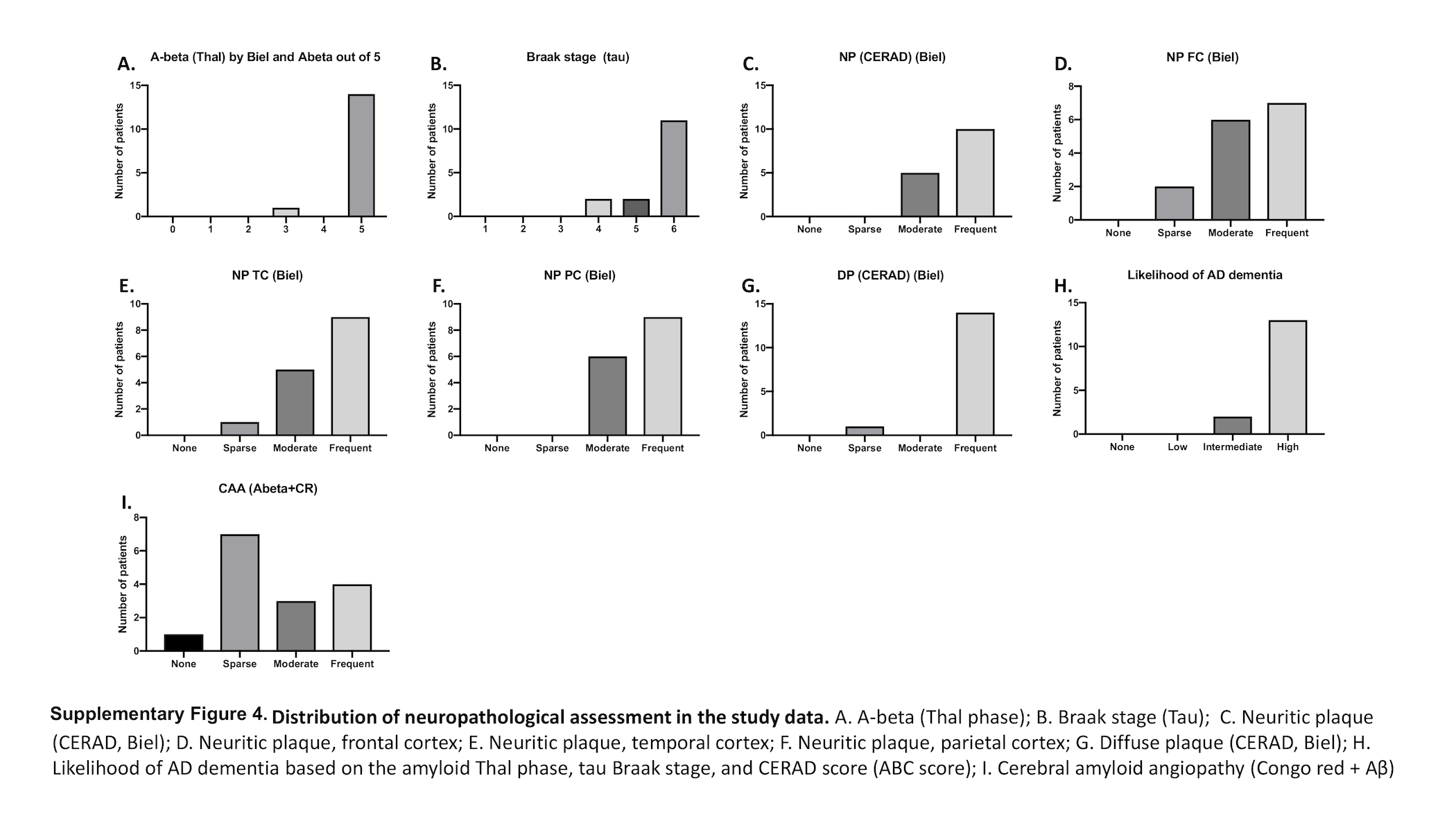

Supplement: FIGURE S4 — Distribution of neuropathological assessment in the study data. (A) Aβ (Thal-phase); (B) Braak stage (Tau); (C) Neuritic Plaque (CERAD, Biel); (D) Neuritic Plaque Frontal Cortex; (E) Neuritic Plaque Temporal Cortex; (F) Neuritic Plaque Parietal Cortex; (G) Diffuse Plaque (CERAD, Biel); (H) Likelihood of AD dementia based on the amyloid Thal phase, tau Braak stage, and CERAD score (ABC score); (I) Cerebral Amyloid Angiopathy (Congo red + Aβ). [file Image_4.tif]

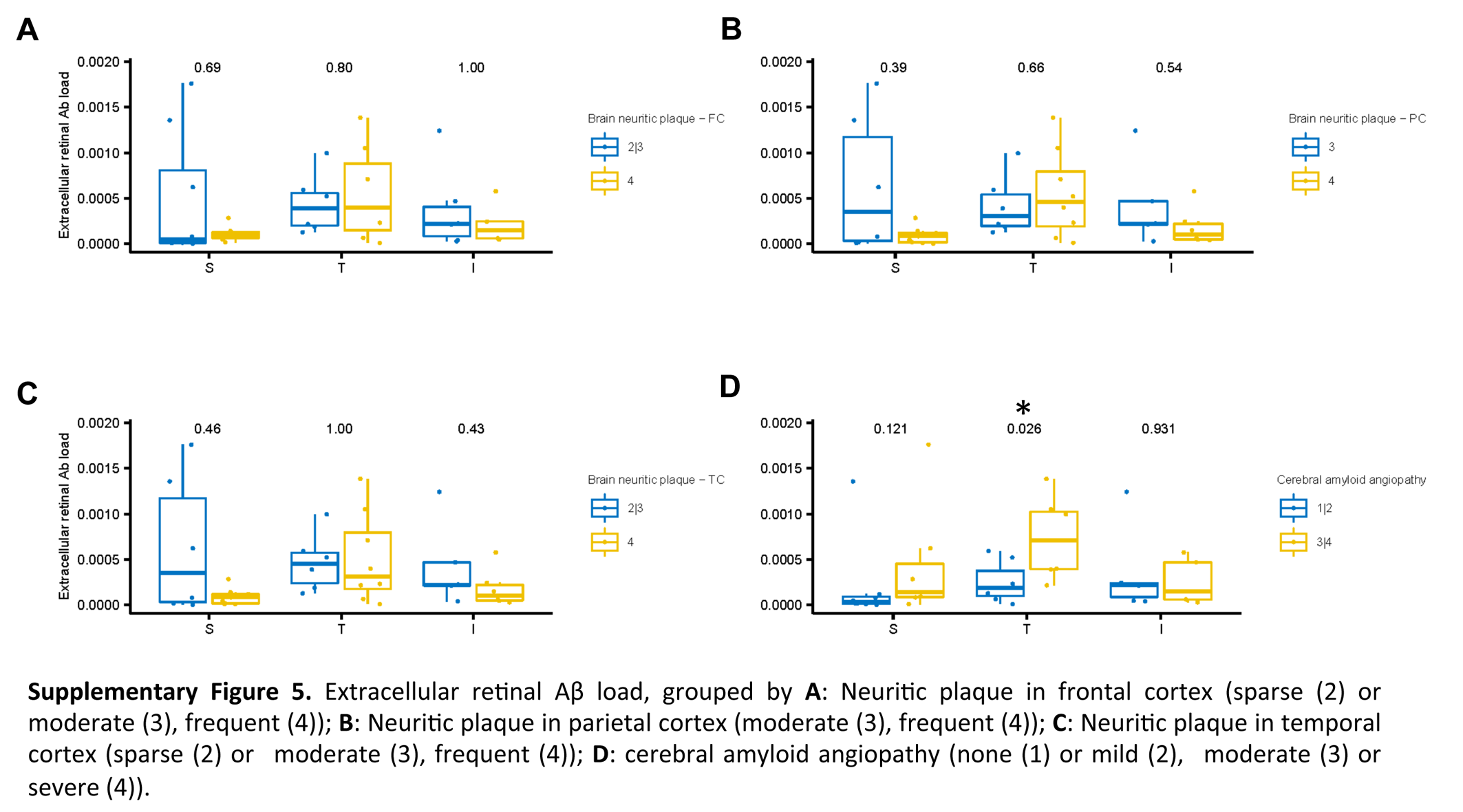

Supplement: FIGURE S5 — Extracellular retinal Aβ load, grouped by (A) Neuritic plaque in frontal cortex (sparse (2) or moderate (3), frequent (4)); (B) Neuritic plaque in parietal cortex [moderate (3), frequent (4)]; (C) Neuritic plaque in temporal cortex [sparse (2) or moderate (3), frequent (4)]; (D) cerebral amyloid angiopathy [none (1) or mild (2), moderate (3) or severe (4)]. [file Image_5.tif]
